# Systemic treatment options for non-small cell lung cancer after failure of previous immune checkpoint inhibitors: a bayesian network meta-analysis based on randomized controlled trials

**DOI:** 10.1186/s12865-024-00633-z

**Published:** 2024-06-28

**Authors:** Kang Wang, Zhenxue Fu, Guanxing Sun, Yancui Ran, Nannan Lv, Enbo Wang, Huan Ding

**Affiliations:** 1 Department of Oncology, Zaozhuang Municipal Hospital, Zaozhuang, 277100 China; 2 Department of Respiratory Medicine, Zaozhuang Municipal Hospital, Zaozhuang, China

**Keywords:** Non-small cell lung cancer, Immune checkpoint inhibitors, Disease progression, Tyrosine kinase inhibitors, Bayesian

## Abstract

**Background:**

Although immune checkpoint inhibitors (ICIs) have brought survival benefits to non-small cell lung cancer (NSCLC), disease progression still occurs, and there is no consensus on the treatment options for these patients. We designed a network meta-analysis (NMA) to evaluate systemic treatment options for NSCLC after failure of ICIs.

**Methods:**

PubMed, Embase, Web of Science and Cochrane Library databases were searched, then literature screening was followed by NMA. We included all Phase II and III randomized controlled trials (RCTs). Progression-free survival (PFS) and overall survival (OS) used hazard ratio (HR) for evaluation. Objective response rate (ORR) and adverse events (AEs) used odds ratio (OR) and relative risk (RR) effect sizes, respectively. R software was applied to compare the Bayesian NMA results.

**Results:**

We finally included 6 studies. 1322 patients received ICI plus Chemotherapy (ICI + Chemo), ICI plus Anti-angiogenic monoclonal antibody (ICI + Antiangio-Ab), ICI plus Tyrosine kinase inhibitor (ICI + TKI), Tyrosine kinase inhibitor plus Chemotherapy (TKI + Chemo), Standard of Care (SOC), Chemotherapy (Chemo). TKI + Chemo is associated with longer PFS, higher ORR (surface under cumulative ranking curve [SUCRA], 99.7%, 88.2%), ICI + TKI achieved the longest OS (SUCRA, 82.7%). ICI + Antiangio-Ab was granted the highest safety rating for adverse events (AEs) of any grade, AEs greater than or equal to grade 3 and AEs of any grade leading to discontinuation of treatment (SUCRA, 95%, 82%, 93%).

**Conclusions:**

For NSCLC after failure of ICIs, TKI + Chemo was associated with longer PFS and higher ORR, while ICI + TKI was associated with the longest OS. In terms of safety, ICI + Antiangio-Ab was the highest.

**Supplementary Information:**

The online version contains supplementary material available at 10.1186/s12865-024-00633-z.

## Introduction

The treatment spectrum of advanced NSCLC has changed greatly in the past few decades.

In the initial stage, the patients mainly relied on chemotherapy, but the benefit was limited. The PFS was only 2.8 months, and 2.9 months of OS was prolonged even if the treatment time was extended [1].

With the discovery of molecular targets, the treatment of lung cancer has entered the era of targeted therapy. Targeted therapy for specific target mutations, such as epidermal growth factor receptor (EGFR), anaplastic lymphoma kinase (ALK), has brought better survival benefits [2–5].

ICIs is a group of drugs designed to improve the effectiveness of treatment for various types of malignant tumors. Both monotherapy and combination therapies have been beneficial as first-line and late-stage treatments [6]. ICIs is also being explored in NSCLC without driver gene or after targeted therapy progression. IMpower110 results showed that atezolizumab significantly improved PFS (HR = 0.63) and OS (HR = 0.59) in wild-type stage IV NSCLC patients with high PD-L1 expression when compared with chemotherapy [7]. The KEYNOTE-042 study further expanded the inclusion criteria to PD-L1 Tumor cell Proportion Score (TPS) ≥ 1% showed that pembrolizumab significantly reduced the risk of death by 19% compared with chemotherapy [8]. Currently, programmed cell death ligand 1 (PD-L1)/ programmed cell death protein 1 (PD-1) inhibitors have become the first-line standard treatment for stage IV NSCLC without driver mutations. Similarly, for advanced NSCLC harboring EGFR sensitive mutations who progressed after EGFR-TKI treatment, ORIENT-31 showed that compared with chemotherapy, sintilimab plus chemotherapy significantly prolonged the median PFS [9]. Our previous NMA showed that ICIs + Chemo + Antiangio was superior to chemotherapy in terms of PFS, OS and ORR for EGFR-mutated NSCLC that progressed after EGFR-TKI, and could be used as the preferred treatment. Even if the ICIs monotherapy, whose OS is better than that of chemotherapy, which can also be used as a treatment option [10]. In second-line therapy, the CheckMate-078 study showed that Nivolumab significantly prolonged OS and increased ORR compared with chemotherapy [11].

Immunotherapy for NSCLC with boundless prospects. However, nearly 70% of patients with advanced NSCLC do not derive lasting clinical benefit from immunotherapy, or some patients have primary resistance to ICIs [12], subsequent treatment is still controversial, unable to reach a consensus. Therefore, we conducted this Bayesian NMA to investigate treatment options for NSCLC after failure of previous ICIs, hoping to provide references for clinical practice and future research.

## Materials and methods

### Registration

Our NMA follows the Preferred Reporting Items for Systematic Reviews and Meta-Analyses 2020 statement for NMA (Supplement 1 in Appendix S1) while applying in the Prospective Register of Systematic Reviews (ID: CRD42023473695).

### Search strategies and inclusion, exclusion criteria

We searched for papers published before October 25, 2023 in four databases (PubMed, Embase, Web of Science and Cochrane Library) (Supplement 2 in Appendix S1). The database retrieval was done independently by KW and ZxF.

Inclusion criteria: (1) Unresectable locally advanced or metastatic NSCLC. (2) Prior treatment with PD-L1/PD-1 or cytotoxic T lymphocyte antigen-4 (CTLA-4). (3) The disease has progressed. (4) RCTs. (5) English full-text articles or conference abstracts. (6) The research results include PFS, OS, ORR, AEs, which can be directly obtained in the literature.

Exclusion criteria :1. Single arm. 2. Phase Ia clinical trial. 3. Experimental design scheme. 4. Case report and review. 5. Insufficient data. 6. Non-RCTs and retrospective studies.

### Data extraction

GxS and NnL separately used spreadsheets for data extraction. In case of disagreement, a third party (HD) shall negotiate and reach an agreement. The baseline characteristics included in the study were: Study (Author, Name, Year, Phase), Clinical Trial Gov. No., Publication Type, Race/ Ethnicity, Mean Age (years), Study Period, Total Patients (Male/Female), Treatment Line Number, Groups, No. Patients, Median Follow-up Months. OS and PFS were evaluated using HR and their standard error (SE). ORR and AEs were evaluated using OR and RR, respectively.

### Risk of bias assessment

We used the modified Cochrane Risk Bias Tool (RoB 2) to evaluate the quality of included studies [13]. Five items were included: risk of bias arising from the randomization process, owing to deviations from the intended interventions, missing outcome data, in the measurement of the outcome and in the selection of the reported result. The results of each evaluation were “low”, “high” or “some problems”. The study was judged to be “low” if all items had low risk, “high” if any of them had high risk, and “some problem” otherwise.

### Data statistical analysis

Application PFS (starting from the research to the disease recurrence time, or the researchers defined), OS (starting from the research to the full by the time of death, or the researchers defined), ORR (objective response percentage, or the researchers defined) to assess the efficacy, and safety is according to Common Terminology Standards for Adverse Events (CTCAE) standard to define, includes any level of AEs, greater than or equal to level 3 AEs, and any AEs that results in discontinuation of treatment. Using SUCRA to evaluate the final ranking. Perform Bayesian NMA using R software (Appendix S2). The *I*^2^ statistic evaluated the heterogeneity of treatment effect between studies. Random-effects model was used if *I*^2^ ≥ 50%, Otherwise, fixed effect model was adopted. Choose a subgroup analysis.

## Result

### The baseline characteristics

**Fig. 1:**
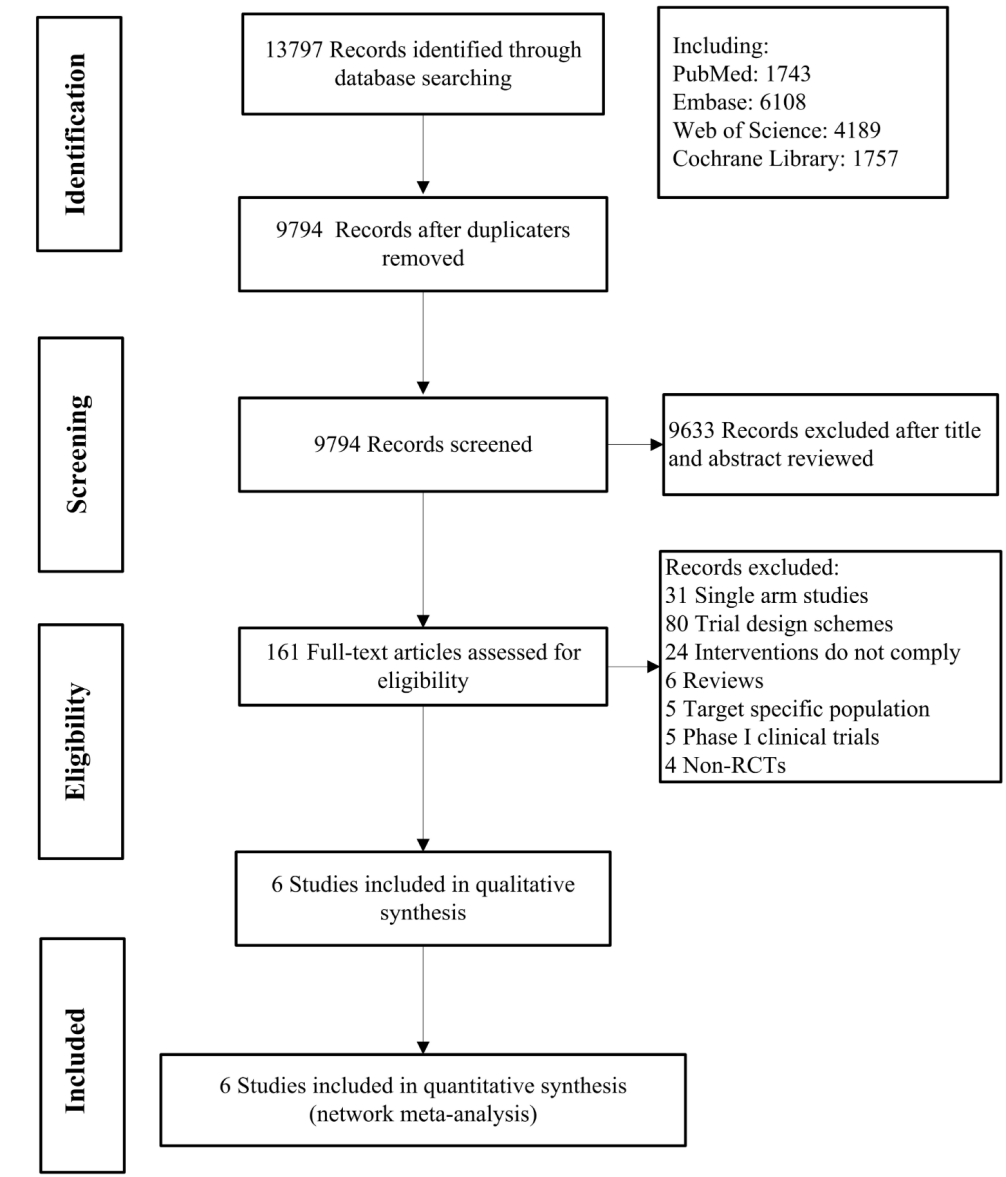
Literature search and screening

Initially 13,797 records were retrieved from the database, and 161 records were further read in full text detail. Ultimately, 6 studies met our criteria (Fig. 1) [14–19]. The sample size ranged from 72 to 577, and total of 1322 participants were included. According to the mechanism of drug action, we divided the treatment regimen into 6 groups, which were: ICI + Chemo, ICI + Antiangio-Ab, ICI + TKI, TKI + Chemo, SOC, Chemo. ICI includes Atezolizumab, Pembrolizumab, Nivolumab. Chemo includes Docetaxel, Chemotherapy (not specific). Antiangio-Ab includes Bevacizumab and Ramucirumab. TKI includes Anlotinib, Sitravatinib and Cabozantinib. SOC: Docetaxel/Ramucirumab, Docetaxel, Gemcitabine, and Pemetrexed (Table 1).

**Table 1:** Baseline characteristics of studies included in the NMA

Study (Author, Name, Year, Phase)	Clinical Trial Gov. No.	Publication Type	Race/ Ethnicity	Mean Age (years)	Study Period	Total Patients (Male/Female)	Treatment Line Number	Groups	No. Patients	Median Follow-up Months
F. Ghiringhelli et al., MORPHEUS-lung, 2022, II	NCT03337698	Conference abstract	NG	63	2018.01.02 - 2022.08.24	72 (52/20)	Second lines and above	Atezolizumab and Bevacizumab	40	NG
								Docetaxel	32	
Hyun Ae Jung et al., 2022, II	NCT03656094	Full text	NG	63 (36–82	2018.11 – 2020.11	98 (80/18)	Three lines and above	Pembrolizumab + Chemotherapy	47	10.5 (8.9-12.1)
				64 (38–79)				Chemotherapy	51	
H. Pan et al., ALTER-L016 and ALTER-L018, 2022, II	NCT03726736 NCT03624309	Conference abstract	NG	62 (31-74)	2018.12.21 - 2022.04.30	73 (64/9)	Second lines and above	Anlotinib + Docetaxel	43	NG
				60 (41-73)				Docetaxel	30	
Karen L. Reckamp et al., Lung-MAP S1800A, 2022, II	NCT03851445 NCT03971474	Full text	White Black Asian or Native American Multiracial Other	66.4 (37.6-85.3	2019.05 - 2020.11	136(83/53)	Second lines and above	Pembrolizumab + Ramucirumab	69	17.9 (1-30)
				65.8 (45.6-84.3)				Standard-of-care (SOC) *	67	
H. Borghaei et al., SAPPHIRE, 2023, III	NCT03906071	Full text	White Black or African American Asian Other	65	2019.07.15 - 2023.03.20	577 (338/239)	Second lines and above	Sitravatinib + Nivolumab	284	17 (16-18.4)
								Docetaxel	293	
J. Neal et al., CONTACT-01, 2023, III	NCT04471428	Conference abstract	NG	64	2020.10.01 - 2022.09.28	366 (NG/NG)	Second lines and above	Atezolizumab + Cabozantinib	186	10.9
				66				Docetaxel	180	

*SOC: investigator’s choice of docetaxel/ramucirumab, docetaxel, pemetrexed or gemcitabine

Since chemotherapy is the most common control group in RCTs and the standard treatment for NSCLC progressing with ICIs, all treatments were compared with chemotherapy.

### Risk of bias

The study results are shown in Supplement 3 in Appendix S1.Data from three studies by J. Neal, H. Pan, and F.Ghiringhelli were derived from conference abstracts and could not assess the risk of bias in the selection of the reported result, so be evaluated as “some concern”. The other three studies were rated as low risk.

### Heterogeneity assessment

We performed heterogeneity analysis for each variable and found high heterogeneity (*I*^2^ ≥ 50%) in the comparison of the following results: Chemo vs. ICI + TKI (84.0%) in PFS and Chemo vs. ICI + TKI (87.3%) in greater than or equal to grade 3 AEs. The heterogeneity of other comparison groups was low.

### Comparison of efficacy indexes PFS, OS and ORR

#### PFS

**Fig. 2:**
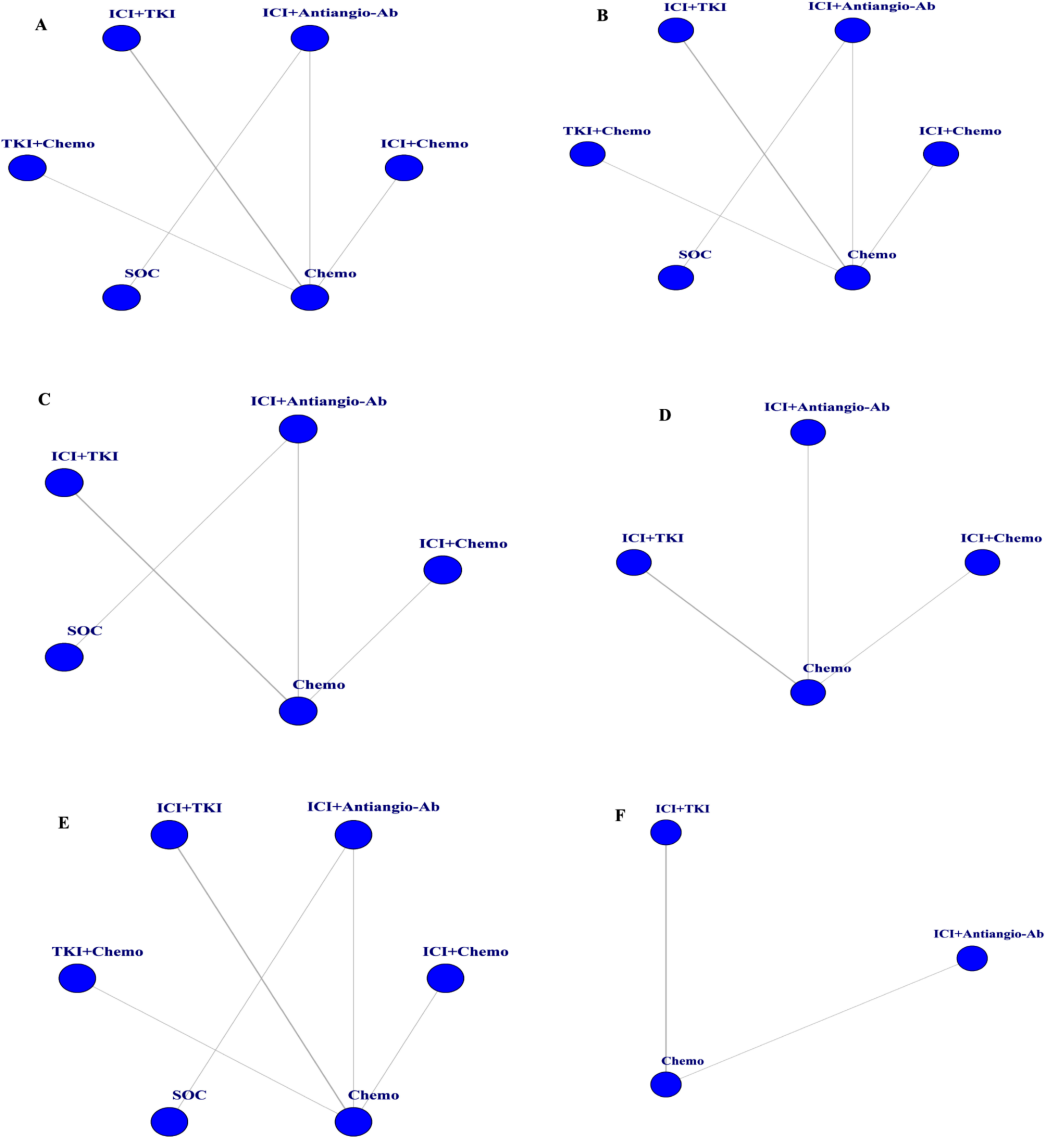
Comparative network plots for NSCLC after failure of previous ICIs. A Bayesian framework is used for comparison. (**A**) PFS. (**B**) ORR. (**C**) OS. (**D**) Safety assessed according to AEs of any-grade. (**E**) Safety assessed according to AEs of grade greater than or equal to 3. (**F**) Safety assessed according to AEs of any grade leading to treatment discontinuation occurred. Each circle represents a treatment. ICI: Immune checkpoint inhibitor, ICI+Chemo: ICI plus Chemotherapy, ICI+Antiangio-Ab: ICI plus Anti-angiogenic monoclonal antibody, ICI+TKI: ICI plus Tyrosine kinase inhibitor, TKI+Chemo: Tyrosine kinase inhibitor plus chemotherapy, SOC: Standard of Care, Chemo: Chemotherapy. PFS: Progression-free survival, OS: Overall survival, ORR: Objective response rate, AEs: Adverse events

**Fig. 3:**
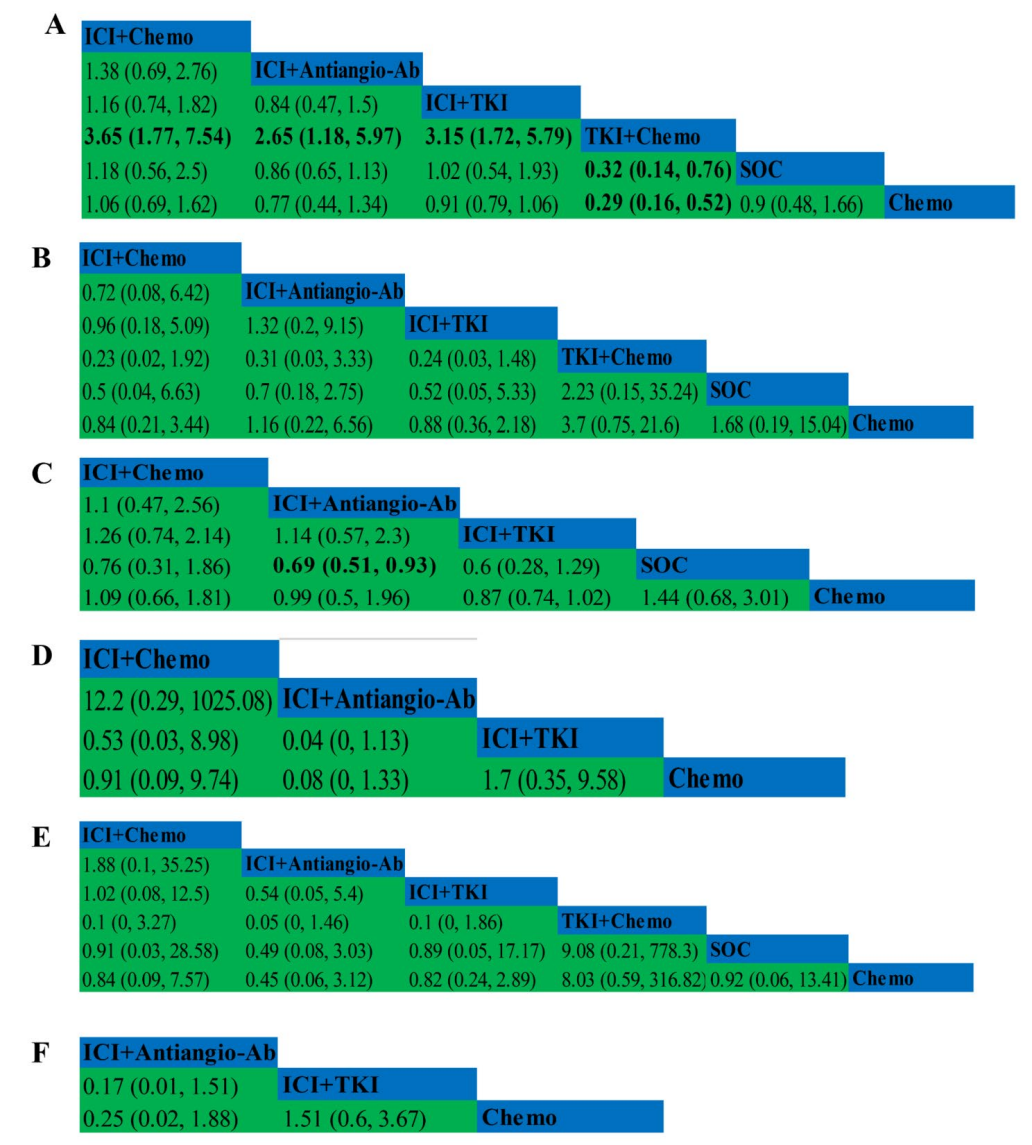
Pooled estimates of the network meta-analysis. (**A**) Pooled HRs (95% CI) for PFS. (**B**) Pooled ORs (95% CI) for ORR. (**C**) Pooled HRs (95% CI) for OS. (**D**) Pooled RRs (95% CI) for Safety assessed according to any grade AEs. (**E**) Pooled RRs (95% CI) for Safety assessed according to grade 3 or higher AEs. (**F**) Pooled RRs (95% CI) for Safety assessed according to any grade leading to treatment discontinuation occurred AEs. ICI: Immune checkpoint inhibitor, ICI+Chemo: ICI plus Chemotherapy, ICI+Antiangio-Ab: ICI plus Anti-angiogenic monoclonal antibody, ICI+TKI: ICI plus Tyrosine kinase inhibitor, TKI+Chemo: Tyrosine kinase inhibitor plus chemotherapy, SOC: Standard of Care, Chemo: Chemotherapy. PFS: Progression-free survival, OS: Overall survival, ORR: Objective response rate, AEs: Adverse events. HR: hazard ratios, OR: odds ratio, RR: relative risk, CI: credible interval

**Fig. 4:**
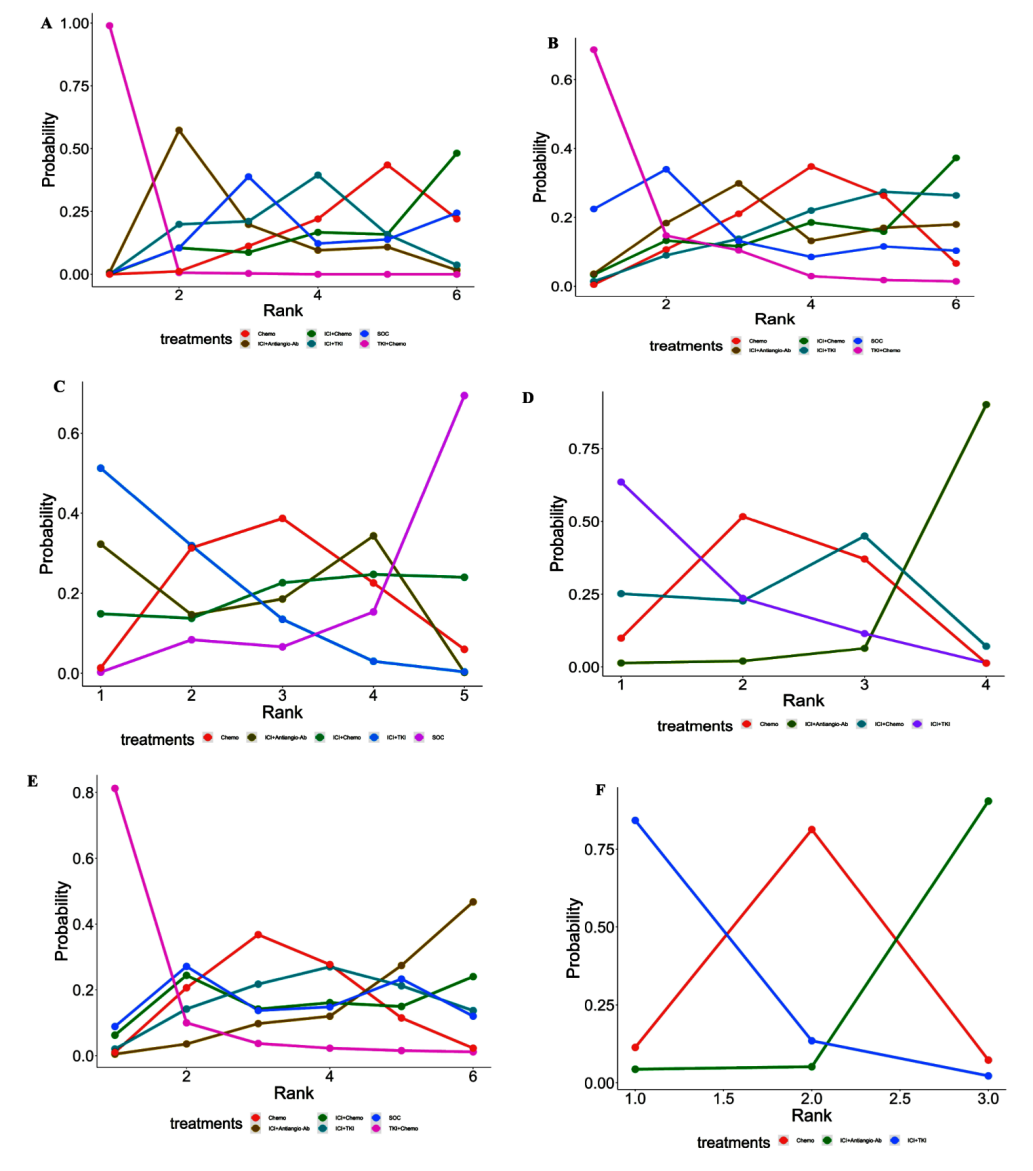
Bayesian ranking profiles of systemic treatment options for NSCLC after failure of previous ICIs. The line graph shows the probability of ranking from first to last for each treatment in terms of PFS, ORR, OS, safety. The abscissa represents “Rank” and the ordinate represents “Probability”. Different interventions are distinguished by different colored lines. The ranking probability for each intervention corresponds to the position of the circle at the ordinate. (**A**) PFS. (**B**) ORR. (**C**) OS. (**D**) Safety assessed according to any-grade AEs. (**E**) Safety assessed according to grade greater than or equal to 3 AEs. (**F**) Safety assessed according to any grade leading to treatment discontinuation occurred AEs. ICI: Immune checkpoint inhibitor, ICI+Chemo: ICI plus Chemotherapy, ICI+Antiangio-Ab: ICI plus Anti-angiogenic monoclonal antibody, ICI+TKI: ICI plus Tyrosine kinase inhibitor, TKI+Chemo: Tyrosine kinase inhibitor plus chemotherapy, SOC: Standard of Care, Chemo: Chemotherapy. PFS: Progression-free survival, OS: Overall survival, ORR: Objective response rate, AEs: Adverse events

Combined treatment outcomes for PFS included 6 studies and 6 treatments (Supplement 4 in Appendix S1) (Fig. 2A). The TKI + Chemo group significantly improved PFS compared with Chemo (HR = 0.29, 95%CI 0.16–0.52), and this benefit was also reflected in the comparison of TKI + Chemo with either group (Fig. 3A). Compared with Chemo, ICI + Antiango-Ab, ICI + TKI, SOC can improve PFS, but the difference was not statistically significant. Ranking analysis based on SUCRA scores shows that TKI + Chemo (SUCRA, 99.7%) is most likely to be the best option for PFS to benefit, followed by ICI + Antiangio-Ab (SUCRA, 64.5%) (Fig. 4A) (Supplement 5 in Appendix S1).

#### ORR

6 studies and 6 treatments had reported ORR (Supplement 6 in Appendix S1) (Fig. 2B). Compared with Chemo, ICI + Antiangio-Ab, TKI + Chemo and SOC all improved ORR, but the difference did not reach statistical significance, while neither ICI + Chemo nor ICI + TKI could improve ORR (Fig. 3B). According to SUCRA score ranking analysis, TKI + Chemo (SUCRA, 88.2%) may be the best choice for ORR benefit (Fig. 4B) (Supplement 5 in Appendix S1).

#### OS

NMA included 5 treatments and 5 studies (Supplement 7 in Appendix S1) (Fig. 2C). The relative effect is shown in Fig. 3C. Compared with the Chemo, both ICI + Antiangio-Ab and ICI + TKI could improve OS, although it was not statistically significant. Compared with SOC, any treatment could bring benefit to OS, but only in ICI + Antiangio-Ab achieved statistical significance (HR = 0.69, 95%CI 0.51–0.93). The ranking analysis based on SUCRA score showed that ICI + TKI was the best choice for OS benefit (SUCRA, 82.7%), and it was obvious that SOC could bring the lowest probability of OS benefit (Fig. 4C) (Supplement 5 in Appendix S1).

### Safety and toxicity

For safety and toxicity, AEs of any grade, greater than or equal to grade 3 and any grade leading to discontinuation of treatment were used separately.

For any grade of AEs, NMA included four studies that included four treatment regimens (Supplement 8 in Appendix S1) (Fig. 2D). ICI + TKI increased the incidence of AEs compared with chemotherapy, while ICI + Chemo and ICI + Antiangio-Ab improved safety (ICI + Chemo vs. Chemo, RR = 0.91, 95% CI 0.09–9.74; ICI + Antiangio-Ab vs. Chemo, RR = 0.08, 95% CI 0-1.33), but the differences did not reach statistical significance ((Fig. 3D).

For AEs greater than or equal to grade 3, all clinical trials and treatment groups were included (Supplement 8 in Appendix S1) (Fig. 2E). Compared with the Chemo, AEs incidence was reduced in all treatment groups except TKI + Chemo (RR = 8.03, 95% CI 0.59-316.82) ((Fig. 3E).

In an analysis of AEs of any grade leading to discontinuation of treatment, only 3 clinical trials compared the 3 treatments (Supplement 8 in Appendix S1) (Fig. 2F), ICI + TKI increased the incidence of AEs compared to Chemo, However, ICI + Antiangio-Ab (RR = 0.25, 95% CI 0.02–1.88) had a better safety profile (Fig. 3F).

Based on the SUCRA score, ICI + Antiangio-Ab had the lowest side effects and the highest safety profile regardless of AEs of any grade, greater than or equal to grade 3 and any grade leading to discontinuation of treatment (Fig. 4D, E and F) (Supplement 5 in Appendix S1).

### Subgroup analysis of baseline immune status on OS

NMA included 2 RCTs and 3 treatments. Compared with Chemo, for PD-L1 < 1% subgroups, ICI + TKI improved OS (HR = 0.76, 95%CI 0.58-1), while ICI + Chemo had the opposite effect (HR = 2.06, 95%CI 1.37–3.1) (Supplement 9 in Appendix S1).

### ICI based treatment

In order to evaluate the benefit of ICI continuation after ICI treatment progression, we performed a regrouping, and the results showed that ICI-based therapy resulted in a benefit compared with Chemo, both in terms of PFS and OS (Supplement 10 in Appendix S1).

## Discussion

### Principal findings

We included 6 RCTs, participants included 1322 advanced NSCLC patients who had progressed after ICIs treatment. Our current study shows that :1. TKI + Chemo can be used as the preferred treatment, providing more survival benefits in terms of PFS and ORR, but the greater than or equal to grade 3 AEs should be considered. (2) In terms of OS, ICI + TKI is the best combination. 3. Compared with chemotherapy, ICI based treatment has benefits in both OS and PFS, which is reflected in ICI combined with any treatment except ICI + Chemo.

Immunotherapy has changed the NSCLC treatment landscape. In first-line treatment, ICIs and their combination with chemotherapy can improve PFS and OS when compared with chemotherapy [7, 20–25]. Based on the results of large clinical studies such as CheckMate-078, KEYNOTE-010 and OAK, immunotherapy with PD-1/PD-L1 inhibitors has become a new standard of second-line treatment [7, 11, 26]. However, even though the KEYNOTE-021 study with first-line pembrolizumab combined with pemetrexed and carboplatin achieved an encouraging OS of 34.5 months, it was still an end-point study, so the selection of patients after progression has attracted more and more clinical attention and some exploration has been carried out.

It is necessary to understand the mechanism of immune resistance in order to select more targeted treatment. Firstly, the change of immune microenvironment is an important research direction after drug resistance. Myeloid-derived suppressor cells (MDSCs) are the main immunosuppressive cells in TME, which can promote tumor invasion, angiogenesis and metastasis, and inhibit anti-tumor immunity. Vascular endothelial growth factor (VEGF) secreted by tumor cells can stimulate the accumulation of MDSCs in TME [27]. In the microenvironment, the vascular system is also responsible for multiple functions such as tumor nutrient supply and immune cell transport, and anti-angiogenic drugs are believed to achieve anti-tumor purposes by normalizing tumor blood vessels and reversing VEGF-mediated immunosuppression. A class of immune cells such as tumor-associated macrophages also exist in TME, which release growth arrest-specific protein 6 (GAS6) to binding Tyro3-Axl-MerTK (TAM) receptors, such as TYRO3 and MERTK [28] and then stimulate the release of immunosuppressive cytokines, ultimately promoting immune suppression of TME. Therefore, targeted blocking of TAM receptors may also be a direction for reversing immune resistance [29]. Compared with chemotherapy, ICI + Antiangio-Ab showed benefits in terms of PFS, OS and ORR, although the differences were not statistically significant. However, compared with SOS, ICI + Antiangio-Ab achieved a statistical benefit of OS. Although the selection of SOC group included single agent chemotherapy and combination with ramucirumab, we suspect that the treatment plan of SOC group was not uniform in the selection of regimen which leading to differences in treatment outcomes. A multi-center international retrospective study of NSCLC patients with disease progression following first-line chemotherapy combined with immunotherapy showed that different chemotherapy regimens also caused differences in OS [30]. Aimed at VEGF and TAM receptor drugs in addition to monoclonal antibodies, as well as a class of small molecule tyrosine kinase inhibitors. We also included three TKI drugs in our study, including Sitravatinib, cabozantinib, and Anlotinib. TKI combination therapy also had good survival benefits. Our NMA showed that TKI + Chemo was the best choice with the highest possible benefit in terms of PFS and ORR, while ICI + TKI was the combination with the highest OS benefit, even in the PD-L1 expression level < 1% subgroup. Because the median OS in ALTER-L016 and ALTER-L018 studies has not been reached, it does not mean that TKI plus Chemo does not provide OS benefit, and OS follow-up is highly anticipated. The side effects of TKI should also be considered behind the encouraging results. AEs of any grade and leading to treatment discontinuation were most frequent in the ICI + TKI group, whereas AEs of grade ≥ 3 were more frequent in the TKI + Chemo group.

Whether ICI can be challenged again after immune resistance is still controversial. A retrospective study by Yixing Wang showed that continuation of anti-PD-1 therapy after initial immunotherapy progress did not bring clinical benefit, nor did it increase the occurrence of adverse reactions [31]. In contrast, the retrospective study by Biagio Ricciuti showed an OS benefit with ICIs after immune progression (HR, 0.32; 95% CI, 0.21–0.46; *P* < 0.0001) [32]. We also analyzed the benefit of continuing ICIs, and the results showed that the continued application of ICIs-based anti-tumor therapy improved both PFS and OS when compared with chemotherapy, but the difference did not reach statistical significance. The formulation of clinical protocols needs to take into account the patient’s PS score, side effects, efficacy and other factors, which also provides a treatment option for patients who cannot tolerate or accept chemotherapy in clinical practice.

At present, the exploration of NSCLC treatment after failure of previous ICIs is still ongoing, clinical results continued to be published after our search deadline [33]. For the sake of data credibility, we only included the RCTs, and excluded many retrospective studies, such as our previous international multi-center retrospective study by Auclin E [30] and the study on ramucirumab combined with docetaxel by Dawar, R [34], the study on immunotherapy versus chemotherapy plus immunotherapy by William P. Tompkins [35]. Lung-MAP sub-study S1400F investigated durvalumab in combination with tremelimumab was excluded because it was a single-arm study [36].

More and more attention has been paid to the progression of NSCLC after ICIs treatment. In addition to the currently published clinical trial results, many RCTs are being recruited and conducted [37, 38]. We also look forward to more evidence-based medical evidence to provide more treatment options and survival benefits for these patients.

### Implication

By conducting a NMA with the limited results of RCTs, our study provides an important reference for clinicians when facing NSCLC patients after failure of previous ICIs. To date, this is the first NMA of treatment options for NSCLC with ICIs treatment progression. Although ICI + TKI may be the best combination for OS benefit based on the current data, we believe that TKI + Chemo may be a potential optimal treatment regimen because it can bring more survival benefit in terms of both PFS and ORR. Since the median survival of ALTER-L016 and ALTER-L018 studies has not been reached, it also means a longer survival period. At the same time, we should pay attention to the greater than or equal to grade 3 AEs in TKI + Chemo, clinical selection needs to be comprehensively considered. Compared with chemotherapy, ICIs-based therapy can also bring benefits in PFS and OS, which means the possibility of continued use of ICIs. In this NMA, we also see the therapeutic potential of small molecule TKI, and hope that more clinical trials can be added to this class of drugs in the future, and it is also a research direction of clinical trials.

### Limitations

Our NMA also has limitations. First of all, the selection of treatment regimens for NSCLC after failure of previous ICIs is an important research hotspot. However, at present, there are few RCTs with end point data. We only included 6 clinical trials, and the data are still limited. Second, although we performed subgroup analyses based on PD-L1 status in the population, there were few data available, and half of clinical trials were from conference abstracts that only analyzed OS for the PD-L < 1% (low expression) subgroup. Third, due to limited data, primary and secondary resistance subgroups were not analyzed. Fourth, the OS data of some studies are incomplete, and the effect of TKI + Chemo combination regimen on long-term prognosis cannot be correctly inferred. Fifth, the inconsistency test could not be completed because our treatment regimens did not form a closed loop in the NMA.

## Conclusion

To date, this is the first NMA of treatment options for NSCLC with ICIs treatment progression. Our study shows that ICIs-based combination therapy can provide survival benefit when compared with chemotherapy, especially ICI + TKI can provide more significant OS benefit, and can be used as a treatment option. TKI + Chemo may be the most promising combination regimen for increasing PFS and OS, but the higher incidence of grade ≥ 3 AEs needs to be considered. Our NMA results need to be further confirmed in large RCTs. In addition, we need to track survival data from ongoing clinical trials to better guide clinical practice.

### Electronic supplementary material

Below is the link to the electronic supplementary material.

**Supplementary Material 1:** Appendix S1

**Supplementary Material 2:** Appendix S2

## Data Availability

The data supporting the findings of this study are available in Appendix of this article.
